# Reconstructing eight decades of genetic variation in an isolated Danish population of the large blue butterfly *Maculinea arion*

**DOI:** 10.1186/1471-2148-11-201

**Published:** 2011-07-11

**Authors:** Line V Ugelvig, Per S Nielsen, Jacobus J Boomsma, David R Nash

**Affiliations:** 1 Centre for Social Evolution, University of Copenhagen, Universitetsparken 15, DK-2100 Copenhagen, Denmark; 2 Current address: IST Austria (Institute of Science and Technology Austria), Am Campus 1, A-3400 Klosterneuburg, Austria; 3 Freelance consultant, DK-2840 Holte, Denmark

## Abstract

**Background:**

Fragmentation of terrestrial ecosystems has had detrimental effects on metapopulations of habitat specialists. *Maculinea *butterflies have been particularly affected because of their specialized lifecycles, requiring both specific food-plants and host-ants. However, the interaction between dispersal, effective population size, and long-term genetic erosion of these endangered butterflies remains unknown. Using non-destructive sampling, we investigated the genetic diversity of the last extant population of *M. arion *in Denmark, which experienced critically low numbers in the 1980s.

**Results:**

Using nine microsatellite markers, we show that the population is genetically impoverished compared to nearby populations in Sweden, but less so than monitoring programs suggested. Ten additional short repeat microsatellites were used to reconstruct changes in genetic diversity and population structure over the last 77 years from museum specimens. We also tested amplification efficiency in such historical samples as a function of repeat length and sample age. Low population numbers in the 1980s did not affect genetic diversity, but considerable turnover of alleles has characterized this population throughout the time-span of our analysis.

**Conclusions:**

Our results suggest that *M. arion *is less sensitive to genetic erosion via population bottlenecks than previously thought, and that managing clusters of high quality habitat may be key for long-term conservation.

## Background

While gene flow decreases the differentiation among populations, it may increase genetic diversity within them. Population connectivity is therefore important to maintain overall genetic diversity across small local populations that would otherwise "erode" because of drift [1]. The effects of isolation by distance and reduced local population sizes tend to be most visible at the edges of species ranges, as these fringes go through periods of expansion with founder effects and contraction with bottlenecks [2]. Empirical studies on butterflies show that peripheral populations are indeed less diverse than central populations [3], and experience larger population fluctuations due to less favourable conditions [4]. In addition the breeding system will also affect within-population genetic diversity, with asexual species being least diverse and sexual systems being variably affected by deviations from random mating, which may affect effective population size independent of drift [1].

Endangered species often occur in small isolated populations where demographic and environmental stochasticity impose additional risks of local extinction. This has made some researchers question the role that genetic factors play in driving population extinction [5], because genetic factors are likely to be negligible when population decline occurs rapidly. However, when effective population sizes remain moderate, inbreeding over many generations may have marked fitness effects due to increasing disease susceptibility and inbreeding depression [2,6-8]. This is because purging tends to remove primarily the few deleterious recessive alleles with large negative effects, and hardly affects the more numerous slightly deleterious alleles [6]. Theory indicates [9] and comparative studies across 170 species have shown [10] that a significant proportion of endangered populations/species have reduced levels of genetic variation compared to related non-endangered species, suggesting that genetic factors often play a role in population extinctions [11].

Researchers have traditionally been forced to evaluate present day diversity of endangered populations against other contemporary populations of the same or closely related species. Such studies are valuable, but since populations rarely have identical demographic and environmental histories, precise identification of the factors that caused extant genetic differences remains impossible. Recent technical advances in the extraction and amplification of old DNA have made the large resources of natural history collections (NHC) available for population genetic studies, providing direct and highly relevant reference points for studies of genetic diversity in endangered populations. Particularly taxa with long histories of collection by entomologists, such as beetles, butterflies and hoverflies, have thus become very useful for long term population studies of genetic change over time.

The number of studies utilizing NHC material for evolutionary genetic studies is increasing, and many focus on past and present genetic diversity in endangered populations [12]. Despite the promises these methods hold for conservation genetics, there are also limitations to the use of historical DNA, and special precautions are required in the experimental and the analytical phase of such work. The highly degraded nature of DNA extracted from historical samples, which increases with age, temperature and water content [13,14], generally restricts PCR amplification to short fragments (< 200 bp) thus limiting the choice of genetic markers. Nuclear microsatellite markers have proven useful in this context, as they have short and highly polymorphic amplicons [12]. However, historical DNA is not only of low quality but also occurs in very low quantity, increasing the risk of genotype errors caused by cross contamination, allelic dropout or false alleles. The importance of following standard protocols when working with historical samples can therefore not be stressed enough, and the assessment and reporting of genotype error rates is indispensable in order to validate such datasets [15-17].

**Figure 1:**
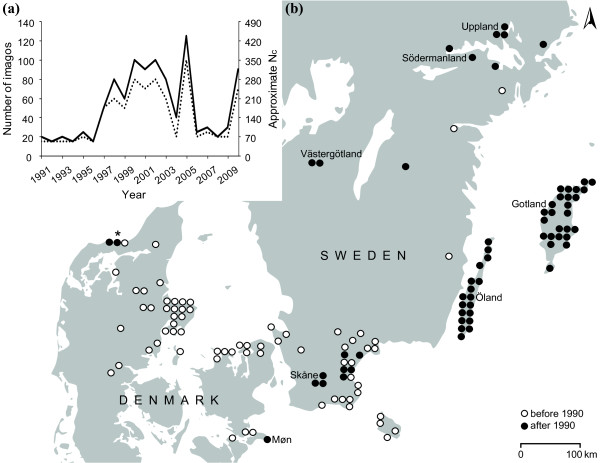
***Maculinea arion *in Scandinavia**. *a*) Count data of *M. arion *imagos on Møn, Denmark. Maximum (solid line) and minimum (dashed line) counts from the best day during the flight season. Imago counts were converted into approximate population census size (# imagos × 3.5) according to Thomas *et al*. [39]. *b*) Distribution of *M. arion *in Denmark and southern Sweden before (open symbols) and after (closed symbols) 1990, 10 km^2 ^UTM grid. Records have been compiled since 1900 by the Atlas Project of Danish Butterflies and the Swedish ArtDatabankens fynddatabas. Danish populations marked by an asterisk went extinct in the late 1990s.

The large blue butterfly, *Maculinea arion*, is one of many butterfly species that have declined in Europe during the last century, both in terms of population numbers and population connectivity [18]. As a result, many extant populations are considered endangered and only exist because they are actively managed [19]. The physical attractiveness and fascinating biology of *M. arion *has made the species popular among amateur collectors, so that many European natural history museums hold large collections often with good numbers of specimens collected in particular years that together form attractive time series for single localities. When these series coincide with periods of population decline they provide an outstanding opportunity to analyse how isolation and demographic fluctuations may have affected genetic variation in the past. Such time series are common for *M. arion *and we exploit such collection material in this study. In particular, we investigated whether/how a recent, severe reduction in population census size and a long history of isolation by distance has affected genetic diversity in the last extant Danish population of *M. arion*, on the island of Møn (Figure 1). As contemporary reference points we used a cluster of six *M. arion *populations in south and central Sweden approximately 100-600 km away [20] and as historical reference points we used NHC specimens from the Møn population covering the time period 1930-1975.

## Results

### Contemporary reference populations

Two of the nine microsatellite loci that were analysed in the contemporary populations departed from Hardy-Weinberg equilibrium after sequential Bonferroni corrections (Møn; Macu17 and Macu20), but without showing evidence of null alleles. Null alleles were found in Macu8, but only in the Møn population and at low frequencies (0.146). Linkage disequilibrium between pairs of microsatellite loci was found between Macu26 and Macu44, and locus Macu26 was subsequently excluded from further analysis.

**Figure 2:**
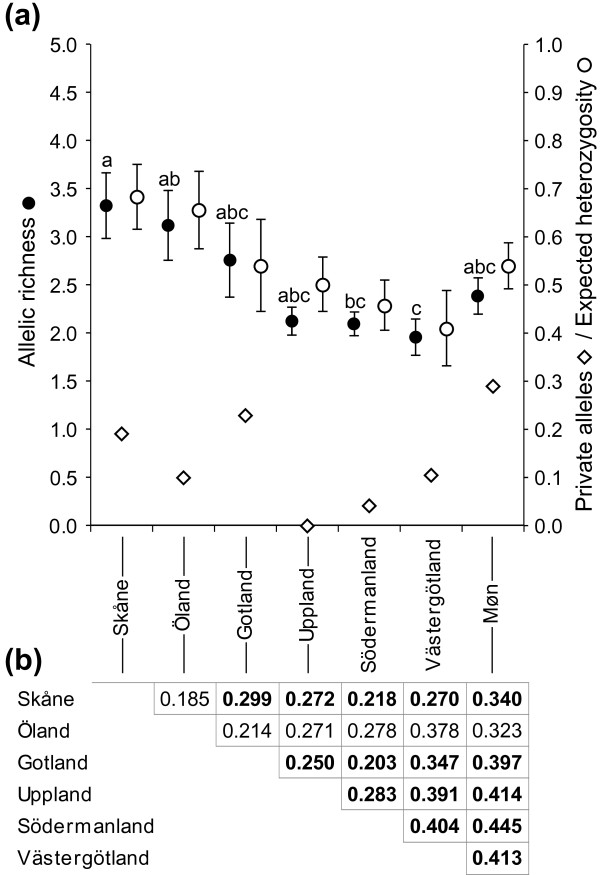
**Genetic diversity and differentiation among Scandinavian *M. arion *populations**. *a*) Two measures of genetic diversity, expected heterozygosity (open circles) and allelic richness (closed circles), were estimated from genotype data of eight microsatellite loci (mean ± SE). Allelic richness differed significantly among populations (One-way ANOVA, *F*_6,55 _= 3.98, *P *< 0.01). Levels not connected with the same letter are significantly different according to post-hoc Tykey-Kramer HSD. Private allele numbers are given as diamonds. *b*) Pairwise genetic distances (*F_ST_*) among the seven contemporary study populations. Figures in bold are significant after standard Bonferroni correction (*P *< 0.05).

The two measures of genetic diversity, allelic richness and expected heterozygosity, differed among the contemporary populations (Figure 2a), but only significantly so for allelic richness (Oneway ANOVA, *F*_6,55 _= 3.98, *P *= 0.004). The Møn population had a medium level of genetic diversity in comparison with the Swedish populations, i.e. lower than the functional metapopulations in Skåne, Öland and Gotland, but higher than three isolated single site populations [20]. The allelic composition was, however, clearly different. While the isolated Swedish populations had few private alleles, the population on Møn had the highest proportion of diagnostic alleles (Figure 2a), even exceeding the numbers found in Skåne, Öland and Gotland. Consequently pairwise *F_ST _*values among the six Swedish populations were lower than their equivalents with the Møn population. The population on Møn was genetically most similar to the geographically closest population in Skåne reflecting a general isolation by distance pattern among population (Mantel's test, *r *= 0.371, *P *= 0.029; Figure 2b).

### Historical reference populations

**Figure 3:**
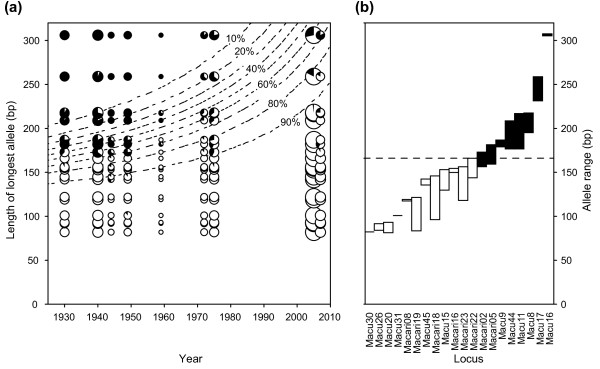
**DNA amplification success in historical samples**. *a*) The longest allele (in base-pairs) amplifying at each locus plotted against the sample age. The size of the symbol indicates the number of samples for which genotyping was attempted at specific loci, and the pie charts give the proportion of samples that successfully amplified (white). Isoclines are given for the amplification success predicted by the GLM model, including sample age and maximum allele length as factors. The amplification success depended on the maximum allele length at the particular locus (*χ*^2 ^= 427.78, df = 1, *P *< 0.0001), the age of the sample (*χ*^2 ^= 54.47, df = 1, *P *< 0.0001) and the interaction between the two (*χ*^2 ^= 51.65, df = 1, *P *< 0.0001). *b*) The 20 microsatellite markers tested in the study and their observed allele ranges. Only loci with amplification success > 80% were used for the temporal study, corresponding to maximum allele sizes below 166 base pairs (below the dashed line).

Only one of 70 DNA extractions of historical samples failed (collection year 1975). Fitting a general linear model to the remaining 69 historical samples revealed that PCR amplification success depended on sample age, the maximum allele length at the specific locus and also the interaction between the two (GLM; sample age: *χ*^2 ^= 54.47, df = 1, *P *< 0.0001; maximum allele length: *χ*^2 ^= 427.78, df = 1, *P *< 0.0001; interaction: *χ*^2 ^= 51.65, df = 1, *P *< 0.0001; Figure 3a). The negative effect of long alleles on amplification success thus increased more than proportionally with sample age. Loci with allele sizes > 166 bp did not amplify consistently in the historical samples (on average 29% amplified, range: 0-100%), and were not used in the further analysis (Figure 3b). Among the microsatellite loci used, genotype error rates per locus were 0.047 on average in historical samples (range: 0.000-0.103) and 0.004 in modern samples (range: 0.000-0.014), an order of magnitude difference (see Additional file 1 Table S1).

Two loci/population combinations departed from Hardy-Weinberg equilibrium after sequential Bonferroni corrections (2005; Macu20 and Macari18). In Macari18 this was due to homozygote excess, and evidence for null alleles was found in four of the sampling years (1940, 1949, 2005 and 2007: frequencies ranging from 0.19-0.25). The loci Macu15 and Macari16 also showed signs of null alleles, but only in sample year 1930 (0.25 and 0.28 frequencies respectively). Due to small sample sizes the presence of null alleles could not be tested for the samples from 1944 and 1959. No linkage disequilibrium was found between any pair of microsatellite loci. Two of the 12 microsatellite loci were monomorphic in all sampling years (Macu30 and Macu31), whereas the remaining ten loci were polymorphic in all populations, except Macari22 in 1944 and Macari16 in 1959.

The two measures of genetic diversity, allelic richness (*F_8,80 _*= 0.326, *P *= 0.954) and expected heterozygosity (*F_8,80 _*= 0.365, *P *= 0.936) did not differ significantly between years, nor between historical and contemporary samples (allelic richness: *F_1,9 _*= 6.87, *P *= 0.133; expected heterozygosity: *F_1,9 _*= 1.82, *P *= 0.402). Furthermore, there was no difference in the genetic diversity measures when comparing all historical samples *vs*. the two contemporary samples. Twelve of the 43 alleles were unique to the historical samples, four of which used to be present at relatively high frequencies (≥0.1; see Additional file 2 Figure S1). In contrast only one allele was unique to the contemporary samples, and was found at lower frequencies (2005: 0.022 and 2007: 0.059; see Additional file 2 Figure S1).

Based on the empirical and simulated *M*-ratios four of the sampling years showed signs of a recent bottleneck, independently of the parameter setting (1930, 1940, 1972 and 2007). The remaining five sampling years showed signs of bottlenecks in the vast majority of parameter combinations, except when the parameters *p_s _*(the fraction of mutations larger than a single step) and *Δ_g _*(the mean size of larger mutations) were set at their maximum (see Additional file 3 Table S2).

**Figure 4:**
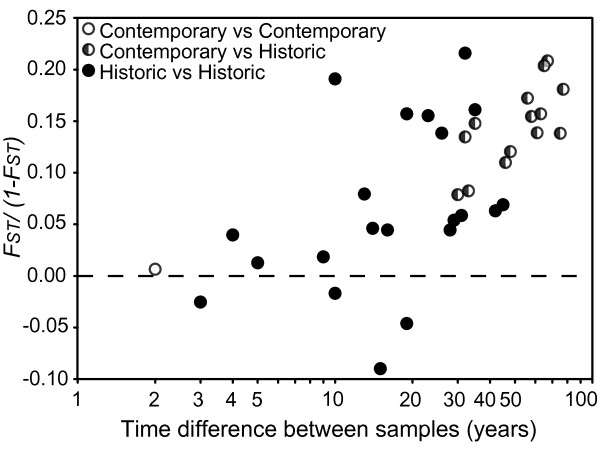
**Temporal genetic change**. The pairwise genetic distance (*F_ST_*/(1- *F_ST_*)), corrected for the presence of null alleles) is positively correlated with the pairwise temporal difference (in years) between sample years at the Møn population (Mantel's test, *r *= 0.647, *P *< 0.01). Comparisons among contemporary (open symbols), historical (closed symbols) and contemporary-historical (half closed) samples are indicated.

The overall *F_ST _*across loci was estimated to be 0.105 (range: 0.024-0.129). The pairwise genetic distances (*F_ST_*) between sampling years were significantly correlated with the temporal difference between samples (Mantel's test, *r *= 0.647, *P *< 0.001; Figure 4), i.e. samples that were fewer years apart were more similar genetically.

## Discussion

### Contemporary vs. historical levels of genetic diversity

We found medium levels of genetic diversity in the *Maculinea arion *population on Møn compared to six contemporary Swedish populations. Significant differences in genetic diversity among populations could only be detected in allelic richness (Figure 2a), which is known to be more strongly affected by population size reductions than is heterozygosity [6]. The very distinct allelic composition of the Møn population shows that ongoing gene exchange with populations in Skåne is restricted, and has been for a long time. This is in line with recent findings showing that gene flow in *M. arion *may occur over long distances (~ 100 km) but only if suitable "stepping-stone" sites are found within *ca *10 km of one another [20]. The fact that the population on Møn is more closely related to the Skåne populations is likely to reflect a shared history, as the open dots on the map in Figure 1b indicate.

While the southern Swedish populations (Gotland, Öland and Skåne) are thought to have been relatively stable over time, with several local populations within dispersal distance [21], the population on Møn has fluctuated markedly in census size in recent decades, and has been through two documented periods of consistently low numbers (Figure 1a). This might imply that the lower levels of contemporary genetic diversity on Møn compared to these southeastern Swedish populations are related to regular moderate bottlenecking. Similarly large fluctuations of *M. arion *have been reported in four UK populations. The magnitude of these oscillation around the carrying capacity has been ascribed to scramble competition between caterpillars after their adoption into the host-ant nests [22, see the Mehod section for a dscription of *Maculinea arion *biology] but the duration of these 'natural' population oscillations are normally shorter than the ones observed on Møn [22], where population census size was reduced to 50-85 individuals over a period of at least six years (Figure 1a).

Despite this, we found no evidence for higher genetic variation in the Møn population prior to the crash around 1991. Levels of allelic richness and heterozygosity did not differ significantly in the studied period covering 77 years (Table 1). Several scenarios may explain this: i) the population reduction was not severe enough to impact the genetic diversity measures. This scenario would be comparable to a study of two species of bumble bees [23] showing that only population reductions by at least 80% resulted in detectable loss of heterozygosity, ii) the number of historical samples was too low to estimate true levels of genetic diversity, or iii) the population was already genetically impoverished before the documented low population size in 1991. We cannot completely exclude that sample sizes in this study may compromise our ability to accurately assess historical levels of genetic diversity, but we believe that the third scenario is the most likely. The exact historical events causing the low level of contemporary genetic diversity on Møn cannot be determined, but the long-term isolation (even prior to 1990, Figure 1b) of the population has undoubtedly had an effect. The populations in northern Sweden show even lower levels of genetic diversity, but analysis of historical data similar to the present study would be needed to reconstruct the causes of these patterns of extant genetic diversity.

**Table 1:** Summary statistics by sampling year for ten microsatellite loci

*Year*	*N*	*k*	*k'*	*H_e_*	*F_IS_*	*M*-ratio
1930	13	33	2.46	0.554	0.151	0.711*
1940	17	33	2.22	0.461	0.136	0.668*
1944	6	26	2.37	0.512	0.191	0.788
1949	9	32	2.36	0.497	0.080	0.743
1959	3	22	2.20	0.480	0.136	0.756
1972	8	27	2.24	0.513	0.228	0.690*
1975	14	29	2.21	0.486	0.132	0.740
2005	46	31	2.38	0.549	0.028	0.757
2007	17	29	2.22	0.491	0.107	0.732*

Given are the sample sizes (*n*) of the years the Høvblege population could be studied and the total number of alleles (*k*), mean allelic richness (*k'*) based on 3 independent diploid individuals, expected heterozygosity (*He*), inbreeding coefficient (*F_IS_*) and the ratio between allele number and allele range (*M*-ratio) calculated according to Garza and Williamson (2001). Asterisk indicates significance level: * *P *< 0.05.

Based on generally accepted measures, only two sampling years (1940 and 1972) showed evidence of bottlenecks, i.e. *M*-ratio < 0.7 [Table 1; but see also 24]. However, when comparing to parameter sets realistic for *M. arion*, all populations showed signs of having passed through a bottleneck except for combinations using extreme parameter values (see Additional file 3 Table S2). This suggests that the Møn population has not been in mutation-drift equilibrium for many decades, and highlights the importance of evaluating *M*-ratio values over a wide range of parameter values when the exact mutation model of the used microsatellite loci is unknown and the population specific *N_e _*uncertain [25].

Although we did not find temporal changes in genetic diversity, 28% of the sampled alleles were unique to historical samples, with half of these present at frequencies above 10% (see Additional file 2 Figure S1). The high number of so-called ghost alleles, i.e. alleles that are lost in modern samples, is remarkable, given the exhaustive sampling in the contemporary population. Ghost alleles are normally reported as evidence of a more diverse past [26-28], which would suggest that the low population census sizes in the early 1990s have had some genetic impact on the Møn population. However, the high temporal turnover in allele frequencies (see Additional file 2 Figure S1) suggests that we cannot rule out that ghost alleles in this study rather reflect the general life history of *Maculinea arion*. The low mobility of patrolling *M. arion *males and the extremely heavy juvenile mortality rates mean that *N_e_*/*N_c _*in *M. arion *is likely to be very low because moderate population bottlenecks occur in each generation. A continuous turnover of alleles possibly associated with occasional female immigrants from far away [29,30] would explain the relationship between pairwise genetic distance and temporal difference between samples (Figure 4).

### Caveats for using historical DNA samples

Studies in the field of conservation genetics increasingly employ NHC samples to assess past genetic diversity levels [12]. Studies of insects have until very recently been underrepresented in these efforts, despite their potential because of large available time series from distinct populations often dating back to the late nineteenth century. While some studies using NHC samples of > 100 years old report successful amplification of nuclear microsatellite loci with alleles exceeding 250 bp [28,31], we found that alleles longer than 160 bp did not amplify consistently in historical samples. Moreover, we found that amplification failure of long alleles increased more than linearly with sample age (Figure 3a), which is in accordance with DNA degradation and fragmentation being random processes, leading to an exponential decline in DNA template with increasing amplicon target size [14,32].

Accordingly, only loci with short allele sizes were used in our present study (Figure 3b), which had the advantage that our genotype error rates (0-10%, see Additional file 1 Table S1) remained at the very low end of the much larger range (0.3-74.6%) reported from other studies using samples of low DNA quantity and quality [33]. This illustrates the importance of assessing the suitability of the genetic markers to be used for such studies, and of reporting genotype error rates that allow independent validation [as advocated by [12], [15], [17]].

## Conclusion

Our study shows that the last *Maculinea arion *population in Denmark is only somewhat genetically impoverished compared to the larger, closest populations in southern Sweden [20]. Contrary to previous opinion, the genetic data of our present study indicate that this pattern is not due to the drastic reduction in population size in the early 1990s, but more likely a consequence of a history of long-term isolation from nearby populations. This emphasises that clusters of interconnected populations are crucial to maintain genetic variation within *M. arion *populations, as the species' extraordinary lifecycle makes local effective population sizes low. For conservation this implies that efforts should not be restricted to the active management of sites currently occupied by *M. arion*, but also include restoration of additional suitable sites within the *ca*. 10 km dispersal range, as is current practice in England and Denmark [34].

While many studies of conservation genetics have a rather pessimistic concluding paragraph, the results of our study indicate that extant *M. arion *populations in northwest Europe may be somewhat more robust than the dismal rates of extinction during recent decades have suggested. The enormous stochasticity of larval mortality likely imposes such consistent effects of genetic drift that local site-specific adaptations have little opportunity to evolve despite relatively low dispersal rates. This may imply that while neutral alleles are turned over at a fairly high rate, there may not be much room for maintaining genetic variation for life history traits that deviate from the species average. The key issue for *M. arion *conservation would thus be active site management to secure optimal conditions for both the specific food-plants and host-ants that large blue butterflies require. Once that has been achieved, the lack of variation for core life history traits may in fact facilitate natural recoveries from low numbers if eventual changes in the local habitat conditions remain within the standard *M. arion *niche. The same characteristics would facilitate re-introduction programs as a source population is unlikely to be differently adapted than the extinct population that it is meant to replace, if they experience similar climatic conditions. Both inferences appear to be consistent with field observations in native and introduced populations [34].

## Methods

### Biology of *Maculinea arion*

*Maculinea arion *is a habitat specialist, like the rest of the genus *Maculinea *(the large blues) and many other members of the tribe Polyommatini (the blues). *M. arion *caterpillars exploit two resources during their development; a specific food-plant on which they feed during the first three weeks after hatching, and subsequently a specific host-ant in the nests of which they live as obligate predators of ant brood, and where they overwinter and pupate in the late spring after 11-23 months [35-37]. This extreme specialization leads to exceedingly high juvenile mortality rates, with 20-40% of a typical population dying in the egg or early larval stages, and mortality among caterpillars inside the host-ant nest representing 80-90% of the total breeding population mortality [22]. Whereas *M. arion *has relatively little impact on the fitness of the food-plant, its main host-ant, *Myrmica sabuleti*, experiences dramatic reductions in colony fitness upon infection [22]. The intimate butterfly-ant relationship leads to large oscillations in census population sizes of *M. arion*, suggesting that genetic diversity may primarily be maintained by gene flow between local low-density populations, rather than substantial effective population sizes at each local site.

### The Møn site and demographic surveys

The number and connectedness of *M. arion *populations in Europe has decreased over the last century. In Denmark *M. arion *was previously known from approximately 40 localities, but only half of these persisted until the second half of the 20^th ^century [Figure 1b; [30]]. At present only a single population remains, at Høvblege on the island of Møn. *M. arion *has a long and probably continuous history of occurrence at this site, with specimens in natural history collections dating back to 1926 (among the earliest in the collections). At that time three to four local populations were found on the island within the typical dispersal distance of the species [max dispersal distance is estimated to be at least 10 km based on genetic markers; [20]].

In 1991 a critically low number of imagos was observed flying at the single remaining Høvblege site, which at this point was *ca*. 8 ha (breeding area). The site, a grassland habitat, had been left almost untouched in the period 1915-1991, allowing for trees, scrub and larger grasses to invade the area with negative consequences for the food-plant distribution. Since 1991 the population has been managed and monitored every year, and now has a breeding area of *ca*. 10 ha. The number of imagos was determined using transect walks, a standard method for assessing year to year changes in butterfly abundance [38]. Population census sizes were estimated according to Thomas [39], assuming that approximately 1/3 of the total population is flying on the best day of the flight season and that 85% of these can be observed during a thorough survey, i.e. *N_c _*≈ 3.5 × the number of imagos counted on the best day. Recent evaluations of butterfly monitoring methods conclude that caution is needed when estimating population census sizes from transect counts [40-43]. The reason being that transect counts are influenced by adult longevity, which is affected by weather patterns and thus vary between years. However, methods such as mark-release-recapture are unfavourable in endangered populations as they may negatively impact the butterflies. Transect counts were therefore consistently used to estimate population sizes in all years, despite yielding cruder estimates.

In 1991-1996 the population size was consistently around 50-85 individuals, but increased to much larger numbers in 1997-2005 (175-440 individuals), to subsequently drop to 70-105 individuals in 2006-2008 (Figure 1a). The lack of empirical data on population size prior to 1991 makes it impossible to estimate the duration and precise magnitude of the population bottleneck around 1991, but according to anecdotal observations the Møn population was very numerous in 1973.

For comparison we used six *M. arion *populations in south and central Sweden (Figure 1b, see Ugelvig *et al*. [20] for details on these sampling localities). Population demography surveys are not available for these populations, but amateur lepidopterists maintain that the populations in Skåne, Gotland and Öland have never been close to extinction. Furthermore, although the number of local populations have also declined in these areas [44], a recent study suggests that well functioning meta-populations still exists at these three sites [20]. Conversely, the populations in Uppland, Västergötland and Södermanland are more isolated [21] and unlikely to still be part of a functioning metapopulation [20].

### Sampling

The contemporary samples were collected in the summers of 2005 (n = 46) and 2007 (n = 17) using a non-invasive sampling technique, collecting 2 × 2 mm^2 ^wingtip fragments from adult *M. arion *butterflies. This sampling technique does not affect survival or flight ability of the butterflies [45; D.R. Nash unpublished data].

The historical samples were kindly provided by the Danish Natural History Museums in Copenhagen and Aarhus, and included specimens collected at Høvblege and the two nearby, now extinct, sites Jydelejet and Møns Klint. Unless there is a need to distinguish them, we will collectively refer to all these sites as Møn. One middle leg per museum specimen was sampled from years in which a reasonable number of specimens were collected, which gave a time series with 4-12 years between samples, covering 77 years (Table 1). After 1975, few *M. arion *specimens exist in the collections, reflecting the rarity of the butterfly, which was finally declared protected in Denmark in 1992. Forceps used for the collection of legs were cleaned in bleach (1% sodium hypochlorite) between each sampling to prevent cross contamination.

### DNA extraction

DNA from the contemporary samples was extracted by homogenizing the wing fragment in a solution of 100 μl 5% chelex-TRIS (10 mM) and 5 μl proteinase K (0.75 units). The samples were then incubated at 56°C for 90 min, boiled at 99°C for 15 min, and centrifuged at 13000 rpm for 3 min. The supernatant was stored at -20°C.

DNA from the historical samples was extracted using a buffer slightly modified from Gilbert *et al*. [46], which consisted of 10 mM Tris (pH 8), 10 mM NaCl, 2.5 mM EDTA, 5 mM CaCl_2_, 2% sodium dodecyl sulphate (SDS), 40 mM dithiothreitol (DTT) and 10% proteinase K (final concentrations). The samples were incubated for 24 h at 56°C with gentle agitation. The extracted DNA was purified using a Qiagen PCR purification kit (QIAquick), re-suspended in 30 μl elution buffer and stored at -20°C. Extraction and PCR-setup was performed in dedicated ancient DNA clean-laboratories at the Centre for GeoGenetics at the Natural History Museum in Copenhagen, where only pre-PCR work occurs. According to standard protocols for work with low quality/quantity DNA [17], contamination was monitored at both the extraction and PCR steps by blank controls and all post-PCR procedures were conducted in physically distant laboratories.

### Microsatellite amplification and genotype error rates

Two sets of nuclear microsatellite markers were employed corresponding to the two research questions (see Additional file 1 Table S1 for details on all microsatellite loci used in the study). In the comparison between contemporary samples from Møn and Sweden nine microsatellite loci were used; Macu8, Macu9, Macu11, Macu15, Macu17, Macu20, Macu26, Macu44 and Macu45 [with genotype data already existing for the Swedish populations, see [20]]. Of these loci, four were suitable for the temporal study, as only loci with allele sizes ≤160 bp allowed amplification in the historical samples (Macu15, Macu20, Macu26, Macu45). Primers for an additional ten loci were developed by ECOGENICS GmbH (Zürich, Switzerland), specifically targeting loci with short allele sizes (< 200 bp); Macu30, Macu31, Macari02, Macari05, Macari08, Macari16, Macari18, Macari19, Macari22, Macari23. Amplification from 1 μl of DNA extracts was carried out in 12 μl mastermix volumes using AmpliTaq Gold (contemporary samples; Applied Biosystems) or Platinium Taq High Fidelity (historical samples; Invitrogen). The following cycling conditions were used: initial denaturation 5 min at 95°C; 35-40 cycles of 30 s at 95°C, 30 s at the locus specific annealing temperature of 56/57°C, 30 s at 72°C; final elongation of 30 min at 72°C. PCR products were run on an ABI 3031 × l automated sequencer with the GeneScan-500 LIZ size standard and analysed using GENEMAPPER 4.0 (Applied Biosystems).

We applied a multiple tube approach when genotyping the historical samples, as they were expected to be prone to genotype errors such as allelic dropout and false allele amplification [16]. The amount of DNA was limited to that extracted from a single butterfly leg, thus it was not possible to replicate as extensively as originally proposed by Taberlet *et al*. (1996; 7-10 replicates per genotype). Instead, two independent amplifications were performed for each sample at each locus. If the same genotype was obtained, this was recorded as the consensus genotype. Conversely, if two different genotypes were found (e.g. one homozygote and one heterozygote) a third PCR was conducted. Genotypes were only scored when every allele was observed at least twice, and in cases where a consensus genotype was not found after three PCRs, it was recorded as missing. Genotype error rates were calculated as recommended by Pompanon *et al*. [15], i.e. the error rate per locus. In the contemporary samples, error rates were estimated by re-genotyping a subset (22%) of the samples.

### Statistical analysis

PCR amplification success was analysed by fitting a general linear model (GLM) with binomial errors and logit link, correcting for over-dispersion, and using the number of samples successfully amplifying at each microsatellite locus as the response variable and the total number of samples as the binominal denominator. The maximum allele length amplified at each locus (in base pairs), the age of the samples (in years), and their interaction were used as explanatory variables. The analysis was carried out in JMP 7.02 (SAS Institute Inc.).

Linkage disequilibrium among pairs of microsatellite loci was tested using FSTAT 2.9.3[47]. The program GENALEX 6.3[48] was used to calculate expected and observed heterozygosities for each microsatellite locus, and for testing genotype frequencies against Hardy-Weinberg (HW) equilibrium expectations. When excess homozygosity was found, the program MICRO-CHECKER 2.2.3 [49] was used to check for evidence of null alleles, and their frequencies at different loci were estimated with the program FREENA[50]. High null allele frequencies were found in some of the historical samples, which may affect F-statistics [50,51]. Pairwise *F_ST _*values among the historical samples were re-calculated after applying the ENA correction for null alleles as implemented in FREENA and then correlated with the temporal difference between samples using Mantel tests (Mantel 1967) in FSTAT, using 2000 permutations. The contemporary populations did not show signs of null alleles, and pairwise *F_ST _*values among the seven Scandinavian populations were calculated in FSTAT, using 1000 permutations. A second measure of genetic diversity, allelic richness, was computed in FSTAT for both contemporary and historical samples and differences in allelic richness and expected heterozygosity among samples were tested using repeated-measures ANOVA in JMP.

Evidence of recent genetic bottlenecks in the temporal samples was tested using the software developed by Garza and Williamson [24]. The program assumes that a reduction in population size will have a stronger affect on the number of alleles (*k*) than the range of allele sizes (*r*), leading to a smaller *M*-ratio (= *k*/*r*) in size-reduced populations compared to equilibrium populations. In order to evaluate the empirical *M*-ratio, an equilibrium population was simulated based on parameters describing the evolution of the analysed microsatellite loci (*Δ_g_*: the mean size of larger mutations, *p_s_*: fraction of mutations larger than a single step, and *μ*: the mutation rate/locus/generation) and the effective population size of pre-bottlenecked populations (*N_e_*). These parameters are difficult to estimate in empirical samples and each sample estimate of *M*-ratio was thus tested under different evolutionary scenarios as suggested by Guinand and Scribner [25]. The scenarios include: i) a two-phase mutation model with proportions of non one-step mutations in the range 0.00 (SMM model), 0.05, 0.10 and 0.20, ii) *Δ_g _*varying between 2 and 4 (Garza and Williamson [24] suggest 3.5 as default setting), and iii) a constant *μ *of 10^-4 ^/locus/generation, but with *N_e _*ranging from 50, 100, 250 and 500 corresponding to *θ *(= 4 × *N_e _*× *μ*) equal to 0.02, 0.04, 0.1 and 0.2. For each sample, an equilibrium population was simulated 10000 times using these parameter settings. The empirical *M*-ratio averaged across loci was compared to the distribution of simulated *M*-ratios, in order to evaluate the likelihood of a bottleneck event having taken place (95% criterion).

## Authors' contributions

LVU carried out the experimental work, and analysed the data together with DRN. PSN contributed with the survey data. The study was designed by LVU, JJB and DRN, who also wrote the manuscript. All authors have approved of the final version of the manuscript.

## Supplementary Material

Additional file 1 **Table S1 - Microsatellite loci used in the study**. Name, GenBank accession numbers, repeat motif and primer sequences (F: forward, R: reverse primer) are given for newly developed microsatellite loci (for previously developed loci see references below). Product size in base pairs and the optimal annealing temperature in degrees Celsius for *M. arion *are also provided. *N *= number of study populations; *n *= number of genotyped individuals; *k *= observed number of alleles; *H*o = observed heterozygosity. The genotype error rate calculated per locus and the fraction of positive PCRs per locus is given separately for historic samples (1930-1975) and contemporary samples (2005-2007). Click here for file

Additional file 2 **Figure S1 - Presence of 'Ghost' alleles in the isolated Danish *M. arion *population**. Allele frequencies per microsatellite locus found in each sampling year. Twelve alleles are only present in the historical samples (black arrows), whereas two alleles are unique to the contemporary (and 1975) samples (white arrows). Microsatellite loci name abbreviations: Ma = Macari, Mu = Macu. Click here for file

Additional file 3 **Table S2 - Detection of recent genetic bottlenecks**. Empirical *M*-ratio values averaged over the ten microsatellite loci for each historic (1930-1957) and contemporary (2005, 2007) sampling year. An equilibrium population was simulated 1000 times for the parameter combination of *Θ*, *Δg *and *ps*, and *P*-values for the occurrence of a genetic bottleneck computed. Grey cells show evidence of a bottleneck (*P*< 0.05), whereas black cells show no evidence of a bottleneck. Click here for file
