# Supplementary material for: Reconstructing eight decades of genetic variation in an isolated Danish population of the large blue butterfly Maculinea arion
Source: BMC Evol Biol. 2011 Jul 11;11:201. doi: 10.1186/1471-2148-11-201 (PMC3146443; doi:10.1186/1471-2148-11-201)
Supplement: Additional file 1 — Table S1 - Microsatellite loci used in the study. Name, GenBank accession numbers, repeat motif and primer sequences (F: forward, R: reverse primer) are given for newly developed microsatellite loci (for previously developed loci see references below). Product size in base pairs and the optimal annealing temperature in degrees Celsius for M. arion are also provided. N = number of study populations; n = number of genotyped individuals; k = observed number of alleles; Ho = observed heterozygosity. The genotype error rate calculated per locus and the fraction of positive PCRs per locus is given separately for historic samples (1930-1975) and contemporary samples (2005-2007). [file 1471-2148-11-201-S1.PDF]

Electronic supplementary material

Reconstructing eight decades of genetic variation in an isolated Danish population of the Large Blue butterfly *Maculinea arion*

L.V. Ugelvig, P.S. Nielsen, J.J. Boomsma and D.R. Nash

Table S1

Marker	Accession	Repeat	Primer seq. (5'-3')	Product (bp)	TA (°C)	*N*	*n*	*k*	*Ho*	Genotype error rate: Historic	Genotype error rate: Contemp.	Positive PCRs: Historic	Positive PCRs: Contemp.
Macari08	HM586088	$(CAA)_8$	F: TAAGTGGGTTCATGGGTGTC<br>R: GCCAGAGGAATTAGCGAGTG	118-119	56	8	131	2	0.285	0.06	0.00	0.90	0.92
Macari16	HM586089	$(TC)_7(AC)_{12}$	F: CCCAGTGTCATAATCCTGTGC<br>R: CTGCATCCCGACAAGACG	150-155	56	8	124	4	0.319	0.07	0.00	0.77	0.95
Macari18	HM586090	$(CA)_{14}$	F: GTGCAATATTCCGGCTTCTG<br>R: CTGCATCCCGACAAGACG	97-145	56	8	131	7	0.225	0.06	0.00	0.90	0.92
Macari19	HM586091	$(TC)_{14}(AC)_8$	F: CGACAAGCATCATGAGTGAG<br>R: CCTATAGTGCGTTTATGCATTG	83-122	56	8	133	5	0.583	0.00	0.01	0.94	0.94
Macari22	HM586092	$(GA)_{11}$	F: TCTGCAGACTGCGAGGTAAG<br>R: TTTTCCTAATTGCGGACTTTC	144-166	56	8	130	5	0.333	0.03	0.00	0.85	0.83
Macari23	HM586093	$(AC)_5(CA)_3AA(CA)_{11}$	F: CGAGGAGATCGAAAATCTATTG<br>R: GGGTGGACATCGAGGATAAAG	118-156	56	8	130	5	0.475	0.10	0.00	0.88	0.85
Macu15	Zeisset *et al.* (2005)			130-153	62	8	129	4	0.370	0.04	0.01	0.88	0.84
Macu20	Ugelvig *et al.* (submitted)			82-93	57	8	133	5	0.507	0.03	0.00	0.93	0.91
Macu26	Ugelvig *et al.* (submitted)			84-92	57	8	131	3	0.564	0.02	0.01	0.90	0.79
Macu45	Ugelvig *et al.* (submitted)			136-142	57	8	121	3	0.384	0.05	0.00	0.77	0.95
Macari02	HM586094	$(CA)_{14}$	F: TCAAGCTTAGCAAATATTCACA<br>R: TTTGGCAACGTGCTTATTAGG	157-173	56	8	92	5	-	-	-	-	-
Macari05	HM586095	$(TC)_7(AC)_{12}$	F: CATCCCCGAGACCTTTCAC<br>R: CGTCATTCTATGATTCCGAAAG	159-182	56	8	75	6	-	-	-	-	-

Macu30	HM586096	$(TG)_{13}$	F: GACGCGCTGTTATGTATTGC<br>R: CGTCTAGCGTGACCGTAACA	82	57	8	133	1	-	-	-	-	-
Macu31	HM586097	$(GTA)_2GTC(GTA)_{17}$	F: GTTCTGTCCCCCGAACTAGG<br>R: AAACCTGGGATTGGTTAAAAAC	101	57	8	133	1	-	-	-	-	-
Macu11	Zeisset *et al*. (2005)			177-217	62	8	73	12	-	-	-	-	-
Macu16	Zeisset *et al*. (2005)			306-308	62	8	38	2	-	-	-	-	-
Macu17	Zeisset *et al*. (2005)			231-259	62	8	56	9	-	-	-	-	-
Macu44	Ugelvig *et al.* (submitted)			177-209	57	8	76	3	-	-	-	-	-
Macu8	Zeisset *et al*. (2005)			195-218	62	8	68	4	-	-	-	-	-
Macu9	Zeisset *et al*. (2005)			179-187	62	8	88	6	-	-	-	-	-
