# Supplementary material for: Reconstructing eight decades of genetic variation in an isolated Danish population of the large blue butterfly Maculinea arion
Source: BMC Evol Biol. 2011 Jul 11;11:201. doi: 10.1186/1471-2148-11-201 (PMC3146443; doi:10.1186/1471-2148-11-201)

Additional file

Reconstructing eight decades of genetic variation in an isolated Danish population of the Large Blue butterfly *Maculinea arion*

L.V. Ugelvig, P.S. Nielsen, J.J. Boomsma and D.R. Nash

Figure S1

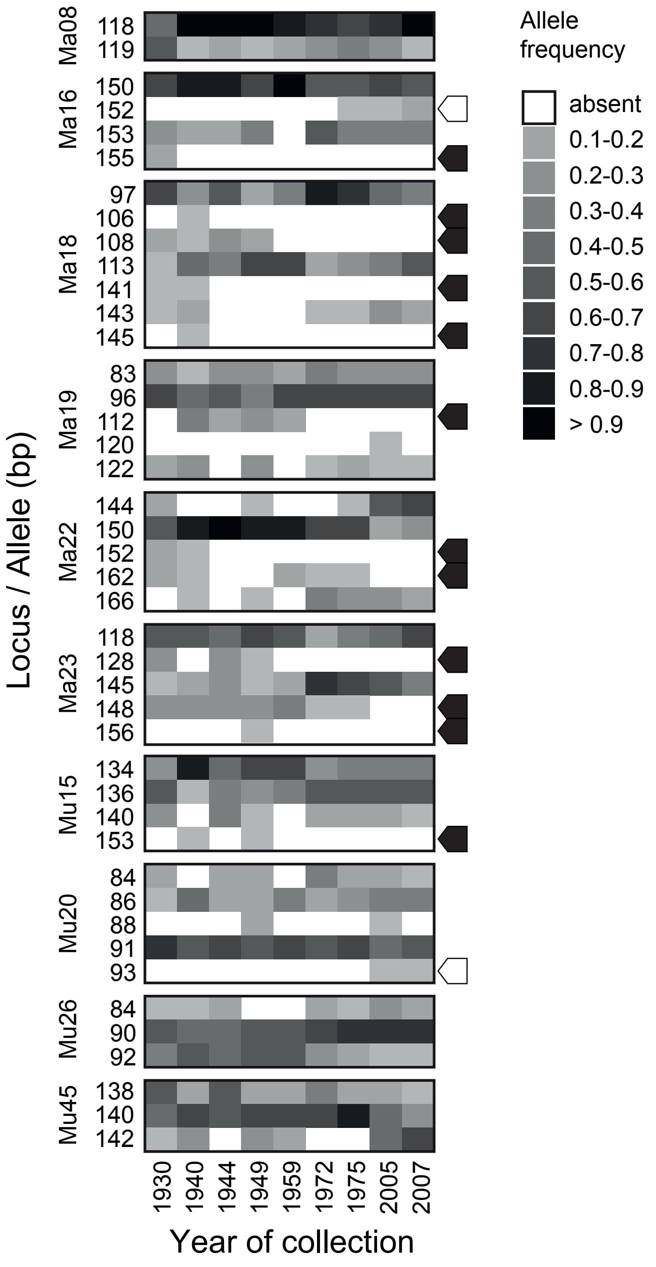

Supplement: Additional file 2 — Figure S1 - Presence of 'Ghost' alleles in the isolated Danish M. arion population. Allele frequencies per microsatellite locus found in each sampling year. Twelve alleles are only present in the historical samples (black arrows), whereas two alleles are unique to the contemporary (and 1975) samples (white arrows). Microsatellite loci name abbreviations: Ma = Macari, Mu = Macu. [file 1471-2148-11-201-S2.PDF]
