# Supplementary material for: Reconstructing eight decades of genetic variation in an isolated Danish population of the large blue butterfly Maculinea arion
Source: BMC Evol Biol. 2011 Jul 11;11:201. doi: 10.1186/1471-2148-11-201 (PMC3146443; doi:10.1186/1471-2148-11-201)
Supplement: Additional file 3 — Table S2 - Detection of recent genetic bottlenecks. Empirical M-ratio values averaged over the ten microsatellite loci for each historic (1930-1957) and contemporary (2005, 2007) sampling year. An equilibrium population was simulated 1000 times for the parameter combination of Θ, Δg and ps, and P-values for the occurrence of a genetic bottleneck computed. Grey cells show evidence of a bottleneck (P< 0.05), whereas black cells show no evidence of a bottleneck. [file 1471-2148-11-201-S3.PDF]

Electronic supplementary material

Reconstructing eight decades of genetic variation in an isolated Danish population of the Large Blue butterfly *Maculinea arion*

L.V. Ugelvig, P.S. Nielsen, J.J. Boomsma and D.R. Nash

Table S2

Population	p_s	$\Delta_g = 2$				$\Delta_g = 3$				$\Delta_g = 4$			
	Θ	0.02	0.04	0.10	0.20	0.02	0.04	0.10	0.20	0.02	0.04	0.10	0.20
1930	0	0.00	0.00	0.00	0.00	0.00	0.00	0.00	0.00	0.00	0.00	0.00	0.00
$M = 0.711$	0.05	0.00	0.00	0.00	0.00	0.00	0.00	0.00	0.00	0.00	0.00	0.00	0.00
	0.1	0.00	0.00	0.00	0.00	0.00	0.00	0.00	0.00	0.00	0.00	0.00	0.00
	0.2	0.00	0.00	0.00	0.00	0.00	0.00	0.00	0.01	0.01	0.02	0.02	0.03
1940	0	0.00	0.00	0.00	0.00	0.00	0.00	0.00	0.00	0.00	0.00	0.00	0.00
$M = 0.668$	0.05	0.00	0.00	0.00	0.00	0.00	0.00	0.00	0.00	0.00	0.00	0.00	0.00
	0.1	0.00	0.00	0.00	0.00	0.00	0.00	0.00	0.00	0.00	0.00	0.00	0.00
	0.2	0.00	0.00	0.00	0.00	0.00	0.00	0.00	0.00	0.00	0.00	0.01	0.01
1944	0	0.00	0.00	0.00	0.00	0.00	0.00	0.00	0.00	0.00	0.00	0.00	0.00
$M = 0.788$	0.05	0.00	0.00	0.00	0.00	0.00	0.00	0.00	0.00	0.00	0.00	0.00	0.00
	0.1	0.00	0.00	0.00	0.00	0.00	0.00	0.01	0.01	0.01	0.01	0.02	0.02
	0.2	0.00	0.00	0.00	0.01	0.04	0.04	0.01	0.07	0.09	0.10	0.11	0.15
1949	0	0.00	0.00	0.00	0.00	0.00	0.00	0.00	0.00	0.00	0.00	0.00	0.00
$M = 0.743$	0.05	0.00	0.00	0.00	0.00	0.00	0.00	0.00	0.00	0.00	0.00	0.00	0.00
	0.1	0.00	0.00	0.00	0.00	0.00	0.00	0.00	0.00	0.00	0.00	0.00	0.01
	0.2	0.00	0.00	0.01	0.00	0.01	0.01	0.01	0.02	0.03	0.03	0.05	0.06
1959	0	0.00	0.00	0.00	0.00	0.00	0.00	0.00	0.00	0.00	0.00	0.00	0.00
$M = 0.756$	0.05	0.00	0.00	0.00	0.00	0.00	0.00	0.00	0.00	0.00	0.00	0.00	0.00
	0.1	0.00	0.00	0.00	0.00	0.00	0.00	0.00	0.00	0.00	0.00	0.01	0.01
	0.2	0.00	0.00	0.00	0.00	0.02	0.02	0.03	0.04	0.04	0.05	0.06	0.08
1972	0	0.00	0.00	0.00	0.00	0.00	0.00	0.00	0.00	0.00	0.00	0.00	0.00
$M = 0.690$	0.05	0.00	0.00	0.00	0.00	0.00	0.00	0.00	0.00	0.00	0.00	0.00	0.00
	0.1	0.00	0.00	0.00	0.00	0.00	0.00	0.00	0.00	0.00	0.00	0.00	0.00
	0.2	0.00	0.00	0.00	0.00	0.00	0.00	0.00	0.01	0.01	0.01	0.01	0.02
1975	0	0.00	0.00	0.00	0.00	0.00	0.00	0.00	0.00	0.00	0.00	0.00	0.00
$M = 0.740$	0.05	0.00	0.00	0.00	0.00	0.00	0.00	0.00	0.00	0.00	0.00	0.00	0.00
	0.1	0.00	0.00	0.00	0.00	0.00	0.00	0.00	0.00	0.00	0.00	0.00	0.01
	0.2	0.00	0.00	0.00	0.00	0.01	0.01	0.01	0.02	0.03	0.03	0.04	0.06
2005	0	0.00	0.00	0.00	0.00	0.00	0.00	0.00	0.00	0.00	0.00	0.00	0.00
$M = 0.757$	0.05	0.00	0.00	0.00	0.00	0.00	0.00	0.00	0.00	0.00	0.00	0.00	0.00
	0.1	0.00	0.00	0.00	0.00	0.00	0.00	0.00	0.00	0.00	0.01	0.01	0.01
	0.2	0.00	0.00	0.00	0.00	0.02	0.02	0.02	0.03	0.05	0.05	0.06	0.08
2007	0	0.00	0.00	0.00	0.00	0.00	0.00	0.00	0.00	0.00	0.00	0.00	0.00
$M = 0.732$	0.05	0.00	0.00	0.00	0.00	0.00	0.00	0.00	0.00	0.00	0.00	0.00	0.00
	0.1	0.00	0.00	0.00	0.00	0.00	0.00	0.00	0.00	0.00	0.00	0.00	0.00
	0.2	0.00	0.00	0.00	0.00	0.01	0.01	0.01	0.01	0.03	0.03	0.04	0.05
